# Employing MCMC under the PPL framework to analyze sequence data in large pedigrees

**DOI:** 10.3389/fgene.2013.00059

**Published:** 2013-04-19

**Authors:** Yungui Huang, Alun Thomas, Veronica J. Vieland

**Affiliations:** ^1^Battelle Center for Mathematical Medicine, The Research Institute at Nationwide Children’s HospitalColumbus, OH, USA; ^2^Department of Biomedical Informatics, University of UtahSalt Lake City, UT, USA; ^3^Departments of Pediatrics and Statistics, Ohio State UniversityColumbus, OH, USA

**Keywords:** linkage analysis, linkage disequilibrium, MCMC, genome-wide association, PPL, PPLD, epistasis, whole-genome sequence

## Abstract

The increased feasibility of whole-genome (or whole-exome) sequencing has led to renewed interest in using family data to find disease mutations. For clinical phenotypes that lend themselves to study in large families, this approach can be particularly effective, because it may be possible to obtain strong evidence of a causal mutation segregating in a single pedigree even under conditions of extreme locus and/or allelic heterogeneity at the population level. In this paper, we extend our capacity to carry out positional mapping in large pedigrees, using a combination of linkage analysis and within-pedigree linkage trait-variant disequilibrium analysis to fine map down to the level of individual sequence variants. To do this, we develop a novel hybrid approach to the linkage portion, combining the non-stochastic approach to integration over the trait model implemented in the software package Kelvin, with Markov chain Monte Carlo-based approximation of the marker likelihood using blocked Gibbs sampling as implemented in the McSample program in the JPSGCS package. We illustrate both the positional mapping template, as well as the efficacy of the hybrid algorithm, in application to a single large pedigree with phenotypes simulated under a two-locus trait model.

## INTRODUCTION

The increased feasibility of whole-genome (or whole-exome) sequencing has led to renewed interest in using family data to find disease mutations. For clinical phenotypes that lend themselves to study in large families, this approach can be particularly effective, because it may be possible to obtain strong evidence of a causal mutation segregating in a single pedigree even under conditions of extreme locus and/or allelic heterogeneity at the population level.

The template for this type of “single large pedigree” design is straightforward. Linkage analysis can be used to narrow the region of interest to a relatively small locus. From there, linkage disequilibrium (LD, or association) analysis can be used for fine-mapping within the linked locus. This step can be based on all sequence variants within the region (whether measured directly in all individuals or partially imputed from selected individuals with sequence and single nucleotide polymorphism (SNP)-chip data in remaining family members). That is, rather than relying solely on bioinformatic filtering approaches to reduce the set of all observed sequence variants down to a manageable number, the set of candidate sequence variants is obtained by (i) restricting the region of interest based on co-segregation with the phenotype, and then within that region, further restricting the set of interesting variants to specific individual mutations co-segregating with the phenotype. Of course, in the presence of appreciable LD among mutations, further filtering and follow-up experiments may be needed to resolve which among a set of correlated mutations is the functional one.

One challenge to this approach is that linkage analysis of large pedigrees is itself not trivial. As is well-known, the Elston–Stewart (ES) algorithm (Elston and Stewart, 1971) can handle relatively large pedigrees, but only a small number of markers at a time. This was less of an issue in the era of microsatellite marker maps, but renders ES relatively ineffective when conducting multipoint analyses using SNPs, because relying on a small number of SNPs per calculation leaves substantial gaps in map informativeness. On the other hand, the Lander–Green (LG) algorithm (Lander and Green, 1987), which can make simultaneous use of large numbers of SNPs, is constrained to smaller pedigrees. Pedigrees with more than around 25 individuals can exceed the limits of the LG algorithm, but these are precisely the pedigrees that can show strong evidence on their own. Trimming or breaking up pedigrees to circumvent LG limitations can lead to substantial loss of information and potentially to misleading results. This is also true of the practice of selecting a small number of affected individuals to use for identity-by-state (IBS) sharing of rare sequence variants, rather than utilizing identity-by-descent (IBD) methods to track variants through the full pedigree structure.

One widely used approach to circumventing the computational complexity of large pedigree calculations is to use statistical methods that avoid calculation of the full pedigree likelihood, such as variance-components (as implemented, e.g., in Almasy and Blangero, 1998). Another familiar alternative is to use Markov chain Monte Carlo (MCMC). This supports the use of the full likelihood, but the difficulties of optimizing performance of samplers tends to limit flexibility in handling the trait model. In particular, we have developed a suite of linkage methods with a very flexible underlying framework for handling the trait model (Vieland et al., 2011) by integrating trait parameters out of the likelihood, one advantage of which is the ease with which new trait models or additional trait parameters can be added to the calculation. MCMC would require separate development and tuning of samplers for each variation of the model, and success in developing well-behaved samplers for all variations is far from guaranteed. For this reason, we have been reluctant to turn to MCMC in the past.

Here we take a novel hybrid approach, combining MCMC to handle the marker data, while retaining the non-stochastic approach to trait–model integration implemented in Kelvin (Vieland et al., 2011). Specifically, we use the graphical-model-based MCMC approach of (Thomas et al., 2000) for the marker data combined with the adaptive numerical integration algorithm described in detail in Seok et al. (2009) for the trait data. This allows us to exploit the power of MCMC in the context of the posterior probability of linkage (PPL) framework (Vieland et al., 2011). We illustrate the application of this new approach by applying it to a single large family.

## MATERIALS AND METHODS

In this section, we (i) present background on Kelvin, the software package in which the PPL framework is implemented, and (ii) on McSample, which implements the underlying MCMC techniques used here. We restrict attention to background directly relevant to this paper (see Vieland et al., 2011 for details on the PPL framework and Thomas et al., 2000 for details on the MCMC methodology). We then (iii) describe the software engineering used to implement the new hybrid method, and (iv) describe the application of the new method to a single large pedigree.

### KELVIN

The PPL framework, as implemented in the software package Kelvin (Vieland et al., 2011), can be used to calculate two primary statistics, both illustrated here: the PPL and the PPLD (posterior probability of linkage disequilibrium, or trait–marker association). The PPL framework is designed to accumulate evidence both for linkage and/or LD and also against linkage and/or LD. All statistics in the framework are on the probability scale, and they are interpreted essentially as the probability of a trait gene being linked (and/or associated) to the given location (or marker). The PPL assumes a prior probability of linkage of 2%, based on empirical calculations (Elston and Lange, 1975), while the PPLD assumes a prior probability of trait–marker LD of 0.04% based on reasoning in Huang and Vieland (2010). This is one caveat to interpretation of the statistics as simple probabilities, since values below the prior indicate evidence against linkage (or LD), while values above the prior indicate evidence in favor. Note too that the small prior probabilities constitute a form of “penalization” of the likelihood; moreover, as posterior probabilities rather than *p*-values, statistics in the PPL framework do not require correction for multiple testing (see, e.g., Edwards, 1992; Vieland and Hodge, 1998 for further discussion of this issue).

One distinguishing feature of this framework is how it handles the trait parameter space. An underlying likelihood in a vector of trait parameters is used. The base models are a dichotomous trait (DT) model parameterized in terms of a disease allele frequency, three genotypic penetrances, and the admixture parameter α of Smith (1963) to allow for intra-data set heterogeneity; and a quantitative trait (QT) model parameterized in terms of a disease allele frequency, three genotypic means and variances corresponding to normally distributed data at the genotypic level, and α. The QT model has been shown to be highly robust to non-normality at the population level and it is inherently ascertainment corrected, so that no transformations of QTs are necessary prior to analysis (Bartlett and Vieland, 2006). Models assuming χ^2^ distributions at the genotypic level are also available to handle QTs with floor effects. The basic QT model can also be extended to cover left- or right-censoring, using a QT threshold (QTT) model (Bartlett and Vieland, 2006; Hou et al., 2012).

Whatever specific model is used, Kelvin handles the unknown parameters of the model by integrating over them for a kind of model-averaging. [Independent uniform priors are assumed for each (bounded) parameter, with an ordering constraint imposed on the penetrances (DT) or genotypic means (QT); see Vieland et al., 2011 for details.]. Kelvin also uses Bayesian sequential updating to accumulate evidence across data sets, integrating over the trait parameter space separately for each constituent data set. This is an explicit allowance for inter-data set heterogeneity with respect to trait parameters, and it also means that the number of parameters being integrated over does not go up with the number of data sets analyzed (see below). A related technique is Kelvin’s use of liability classes (LCs): individuals are assigned to an LC, and the integration over the penetrances or means is done separately for each LC. This is an explicit allowance for dependence of the penetrances (or means) on a classification variable. While current computational restrictions preclude the use of more than three or four LCs at a time, one very important use of this model is incorporation of gene–gene interaction by classifying individual based on their status at a known gene or SNP; we illustrate this approach below.

Due to the nature of the underlying trait models, which are formulated based on genetic considerations without regard to computational convenience, analytic solutions to the resulting multi-dimensional integrals are not possible. Instead, Kelvin carries out the integration over the trait parameters using a modified version of DCUHRE (Berntsen et al., 1997; Seok et al., 2009), a sub-region adaptive or dynamic method, tailored to the specific features of our application. While non-stochastic in nature, the method tunes the amount of resampling of the parameter space to the shape of that space (peakedness) on a position-by-position basis for each data set, resulting in a highly efficient approach to obtaining accurate estimates of the integral. The algorithm is theoretically guaranteed to be accurate for up to 13–15 dimensions, a limit that we generally do not exceed (see above); and because the method is non-stochastic, we do not need to worry about burn-in, convergence or other issues that can complicate Monte Carlo-based approaches.

Kelvin source code is available for download at http://kelvin.mathmed.org/ and Kelvin documentation is accessible on the same site. Help with access, installation, and use can be requested by emailing http://kelvin@nationwidechildrens.org.

### McSAMPLE

McSample is a program for sampling the inheritance states in a pedigree of relatives from the conditional distribution given the structure of the pedigree, observed genotypes and/or phenotypes for individuals in the pedigree, and a model for the founder haplotypes. It is written in Java and is part of the Java Programs for Statistical Genetics and Computational Statistics (JPSGCS) package available from Alun Thomas (http://balance.med.utah.edu/wiki/index.php/Download). The sampling is done using blocked Gibbs updates of two types: ones involving all the inheritance states associated with a locus, and ones involving inheritance states associated with sets of individuals as described by Thomas et al. (2000). Founder haplotype models can be derived under the assumption of linkage equilibrium from the allele frequencies in a sample. It is also possible to estimate models under LD using the FitGMLD program that is also available in JPSGCS, as described by Thomas (2010) and Abel and Thomas (2011). In the case that LD is allowed, only locus block Gibbs updates can be made which typically leads to poorer mixing of the MCMC sampler. The input to McSample must be provided in the format used by the LINKAGE programs (Ott, 1976) with extensions when there is LD. Missing data are allowed in the input. In McSample output, the inheritances are specified by labeling each founder allele uniquely and listing the alleles inherited by each person in the pedigree. There are no missing data in the output. A different output file is created for each iteration. These output files can then be used as input, e.g., to standard lod score calculating programs, with the results averaged over iterations. Note that a standard application would consist of averaging over MCMC-based marker likelihoods for a single, fixed trait model.

### SOFTWARE ENGINEERING

The only difficulty in combining MCMC to handle the marker data with Kelvin’s non-stochastic algorithm for the trait parameter space is one of order of operations. On the MCMC side, calculations are done on a per-pedigree basis for an entire chromosome at a time, and likelihoods are averaged across iterations. For the trait model, however, the adaptive algorithm works by averaging the likelihood ratio (LR, not likelihood; see Vieland et al., 2011 for details) across pedigrees, one calculating position at a time as we walk down each chromosome. Thus there are two iterative processes that need to be decoupled and properly tracked: first, repeated MCMC marker-sample generation for each pedigree across the chromosome; second, repeated (adaptive) trait-space sampling across pedigrees at each position on each chromosome, conditional upon the marker data obtained from the MCMC runs and the trait data. In order to minimize confusion in the exposition that follows, we use “iteration” to describe each individual marker configuration as generated by the MCMC routine in obtaining the marker likelihood, and “trait vector” to describe each individual vector of values for the trait parameters generated by Kelvin to calculate the trait likelihood conditional on the marker information.

To address the required bookkeeping issues while maintaining modular code with minimal changes to existing logic, we adapted Kelvin by simply inserting a set of McSample runs at the beginning of the calculation. At this step, multiple MCMC iterations are generated for each pedigree conditional on the marker data only. Each iteration creates a set of pedigree files with fully informative, phased marker genotypes for each pedigree and each chromosome. We create a single pedigree file incorporating all iterations for each pedigree, with the pedigree label modified to reflect both the pedigree and the iteration. To calculate the LR for a pedigree, we first calculate the LR for each iteration as if it represented a unique pedigree. For each trait vector we average these LRs across iterations for each pedigree at each calculation position along the chromosome, returning a set of LRs by pedigree by position for each trait vector. These LRs are multiplied across pedigrees to obtain the LR by position across pedigrees for each vector, and averaged over all trait vectors. The average LR per position is then evaluated, on the basis of which additional trait vectors may be added in an iterative process until the adaptive trait–model integration algorithm terminates.

The marker likelihood calculation itself is done using the ES algorithm, based on the two markers flanking each calculation position in turn. Because each individual MCMC iteration is fully phased and fully informative, using two markers is equivalent to using all markers with computational complexity no longer a function of the total number of markers. (Indeed a single marker could be used, but because of Kelvin’s built-in algorithm for walking down each chromosome in multipoint analysis, three-point calculations were simpler to implement.) Trait calculations per position are also done based on the ES algorithm regardless of pedigree complexity (Wang et al., 2007). Thus the overall complexity of the MCMC-PPL analysis is linear to the product of the number of iterations, the number of pedigrees, the number of individuals and the number of trait vectors, the last of which differs across calculating positions.

**FIGURE 1:**
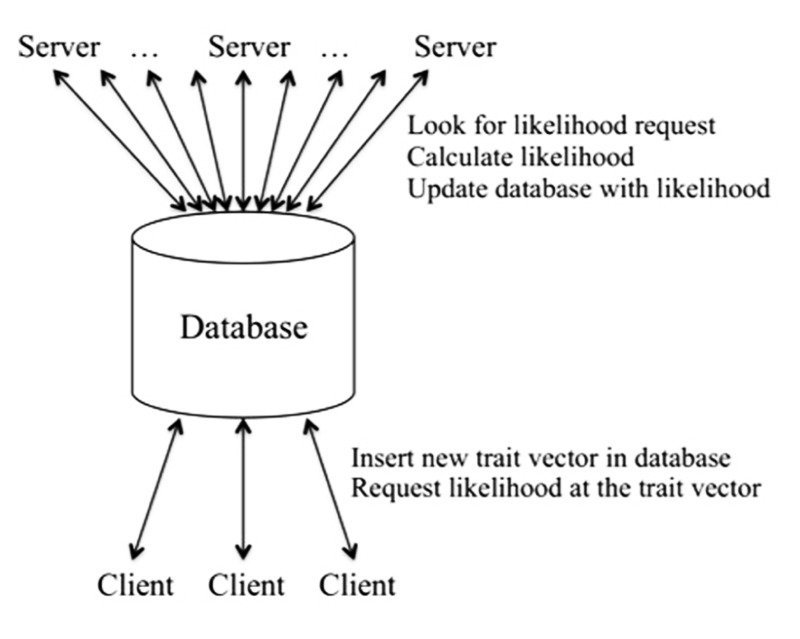
**Client–server architecture in Kelvin**.

In order to decouple the adaptive trait–model integration process from the likelihood calculations, we use the software engineering trick of employing a client–server architecture together with a database to facilitate the operations (see **Figure 1**). The client is the driver for the generation of trait vectors, deciding which trait vectors are needed for the likelihood evaluation at each position, as described in detail in Seok et al. (2009). The client requests likelihoods for the trait vectors from the server using the database as an intermediary. If requested trait vectors are not available in the database, the client adds the required entries to the database for each pedigree for the given calculation position. Once the likelihoods are available for all pedigrees, the client uses them to calculate integrals for the current set of trait vectors and to decide whether additional trait vectors are needed, in which case the process is repeated until the client determines that no additional sampling of the trait vector space is needed.

On the server side, once initiated the server searches the database for trait vector entries flagged as new. It performs the needed likelihood calculations, stores the results in the database, and marks the entry for that trait vector, pedigree, and position as complete/available. Here the server is not a physical node, but rather a likelihood-calculation process. Typically our analyses involve a small number of client processes and many likelihood servers. (Thus this is the reverse of the typical client–server model with a small number of servers and many clients. Nonetheless, our likelihood client plays the usual client role, by sending many requests to the likelihood servers.) The integration process is fast and efficient, requiring very little in terms of computing resources, and for this reason only a few client processes are required. By contrast, the likelihood calculations are highly computationally intensive. Thus the more servers, the faster the overall speed of the analysis. Here the database serves not only as a bookkeeping device, but also as the single server interface to a large pool of server processes.

The client–server architecture supports considerable flexibility in overall Kelvin functionality. It allows us to dynamically add and delete servers as needed. It also allows us to dedicate each server to one pedigree, with the amount of memory and number of cores tailored to the complexity of the pedigree, for efficient use of a distributed computing resource. The client is also by design indifferent as to how the underlying marker likelihood is calculated, i.e., the mechanism used to request and retrieve likelihoods is the same regardless of what approach was used to generate the likelihood. This allows us in principle to mix and match approaches to the marker data, e.g., using the LG algorithm for pedigrees small enough for LG to handle while simultaneously employing MCMC for larger pedigrees, all within the same data set.

### APPLICATION TO SIMULATED DATA

**FIGURE 2:**
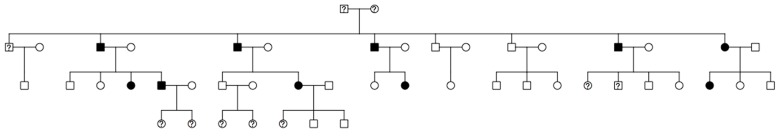
**Structure of the analyzed pedigree (filled, affected; empty, unaffected; ?, unknown phenotype and genotype)**.

To illustrate the use of this new hybrid MCMC–Kelvin approach, we selected a single large pedigree from an ongoing study of real human data. The pedigree has 48 individuals spanning four generations (see **Figure 2**); all but 10 individuals were genotyped. We used actual genotypes for 664,278 SNPs (after comprehensive cleaning) from the Illumina Human OmniExpress 12 V1.0. However, we simulated a new phenotype (for all but the 10 individuals missing genotypes) by selecting two SNPs (rs6851302@178.68cM on chromosome 4, which we call locus 1, and rs1145787@102.65cM on chromosome 6, which we call locus 2), with population frequencies (based on additional data not used here) matching our generating model as specified below; these SNPs were selected additionally for entering the pedigree through the top-most founders and segregating to the next generation at least four times to ensure they would be at least moderately informative in this pedigree.

Phenotypes for each individual were generated assuming an underlying two-locus (2L) disease model based on genotypes at this pair of SNPs. The generating model stipulated disease gene frequency of 1% (locus 1) and 20% (locus 2), and a fully penetrant dominant–dominant (DD) model. This model was selected from a set of 2L models considered in Vieland et al. (1992), which suggested that locus 1 would be moderately easy to map given sufficient meiotic information, while locus 2 might be very difficult to map; the model also represents a major gene effect with a modifier, something we might be interested in studying individual pedigrees. However, the purpose here is not to undertake a comprehensive study of power under different models, but simply to illustrate our approach in application to a single, albeit possibly atypical, pedigree.

Our overall approach to analyzing the pedigree is as follows:

1. We thinned the marker map following standard procedures to eliminate marker–marker LD, after filtering out markers with minor allele frequencies lower than 25%, and applied the new hybrid MCMC–Kelvin method to perform genome-wide linkage analysis. For purposes of this analysis the locus 1 and 2 SNPs were omitted from the marker set analyzed. We based the analysis on 2,000 MCMC iterations combined from 10 independent sampling processes (with different seeds), each with a 1,000-sample burn-in and 200 iterations/sampling run. (See below for rationale.) Linkage calculations were made every 2 cM under Kelvin’s standard single-locus (SL) DT model.
2. We applied the PPLD to fine map under the (primary) linkage peak obtained in the first step, now utilizing all of the available SNPs (including those trimmed out during the first step and the locus 1 and 2 SNPs). While we did not have whole-genome sequence available for this pedigree, if such data were available, then this step would be applied to each variant in turn under the peak(s).
3. We repeated step 1, this time conditioning on genotypes at the most highly associated SNP from step 2, under a 2L model. Specifically, we assigned each individual to a LC based on the individual’s SNP genotype. Kelvin then integrates over the trait parameters separately within LC as described above, which allows for dependence of penetrances on LC. We rescanned the genome under this model in order to look for possible modifier loci interacting with the gene under the primary linkage peak. We also carried out conditional 2L-PPLD analyses to see if we could fine map under a secondary linkage peak down to the level of the individual modifier SNP (or sequence variant, if we had sequence available).

In addition to these analyses, we also used the simulated pedigree to assess variability of the MCMC portion of the calculations. First, we repeated the entire MCMC process as described above five times, and examined variability of the results across these five runs. Second, we ran a single, much longer sampling process (20,000 iterations) for which convergence was almost certainly achieved, then compared our results as described in step 1 with the final 5,000 iterations from the tail (post-convergence) end of this run. Finally, we considered variability across individual runs of 200 iterations with a 1,000-sample burn-in, that is, the length of runs that were averaged over in step 1 above.

## RESULTS

In this section we (i) show results of the analysis of the single large pedigree. We then (ii) consider the accuracy of the MCMC component of the analysis.

### DATA ANALYTIC RESULTS

**FIGURE 3:**
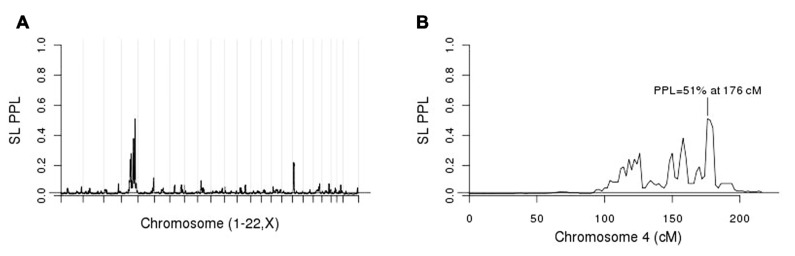
**(A)** Single-locus (SL) genome scan; **(B)** chromosome 4 alone.

**Figure 3A** shows the initial linkage scan. A peak on chromosome 4 clearly stands out above background noise, and we considered this to be our primary linkage finding. The PPL is elevated across a broad region of the chromosome (**Figure 3B**). However, the strongest evidence of linkage spans a relatively short region at approximately 175–181 cM.

For purposes of fine-mapping, we considered any positions on this chromosome with PPL ≥ 10%. The resulting (non-contiguous) region contained 9,433 SNPs from the full original marker set. Forty-nine percent of the analyzed SNPs within the linked regions gave evidence against LD (PPLD < 0.0004), while only six SNPs (0.064%) showed PPLD ≥ 5% (**Table 1**). Two SNPs (rs6851302 and rs654089) clearly stand out from the rest, with PPLD = 0.43 in both cases. These two are in complete LD with one another (*R*^2^ = 1) and in fact they share the same genotypes across this pedigree; rs6851302 and rs11934037 also show some LD (*R*^2^=0.28). Note that even had we restricted fine-mapping to just the best supported region (175–181 cM), we would have successfully found this LD peak. Also for reference purposes, had we selected all 15,531 SNPs from all regions across the entire genome with PPL ≥ 10%, only one additional SNP would have given PPLD ≥ 5% (rs9916791, at 21.73 cM on chromosome 17, PPLD = 0.05).

**Table 1:** Chromosome 4 SNPs with PPLD ≥ 5%.

Chromosome	SNP	cM	BP	PPLD
4	rs1800792	157.60	155753857	0.07
4	rs11100000	158.54	156542439	0.1
4	rs1460128	158.54	156544989	0.09
4	rs11934037	178.57	176255309	0.06
4	rs6851302	178.68	176328488	0.43
4	rs654089	178.71	176347501	0.43

**FIGURE 4:**
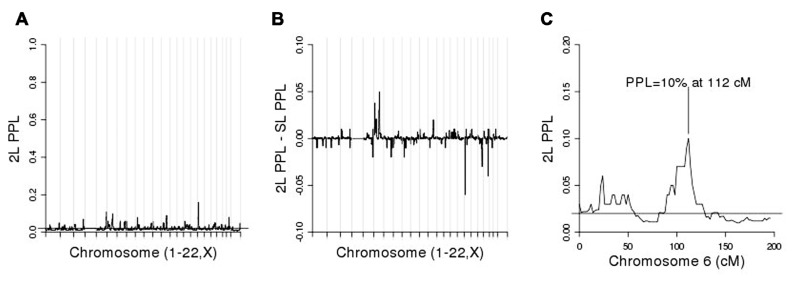
**(A)** Two-locus (2L) genome scan; **(B)** 2L-PPL – SL-PPL across the genome; note that the scale of the *y*-axis is [-0.1, 0.1]. **(C)** Chromosome 6 alone.

We then conditioned on rs6851302 in order to rescan the genome for evidence of modifier loci. (Clearly choosing to use rs11934037 instead would yield identical results.) **Figure 4A** shows the 2L genome scan and **Figure 4B** shows the difference between the 2L and SL-PPLs across the genome (a measure of how much the data “prefer” the 2L model over the SL model). There are no large 2L peaks (**Figure 4A**). However, using the difference between the 2L and SL-PPLs as an indication of how much the data “prefer” the 2L model over the SL model (**Figure 4B**), the largest positive difference occurs on chromosome 6 at 112 cM (SL-PPL = 5%; 2L-PPL = 10%). The doubling of the PPL under the epistasis model suggested a possible modifier gene location. We determined the width of the linkage peak by visual inspection as covering approximately 100–114 cM (see **Figure 4C**), and ran conditional 2L-PPLD analyses on all 3,120 SNPs in this region.

**FIGURE 5:**
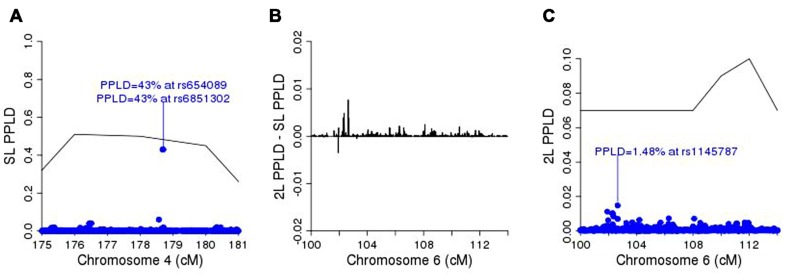
**(A)** SL-PPLD under linkage peak region on chromosome 4 with solid line depicting PPL; **(B)** 2L-PPLD – SL-PPLD across linked region on chromosome 6; **(C)** 2L-PPLD under the 2L linkage peak on chromosome 6.

**Figure 5A** shows the SL-PPLD under the linkage region on chromosome 4, and **Figure 5B** shows the 2L-PPLD – SL-PPLD across the linkage region on chromosome 6; again a single region is elevated in the 2L analysis, with the highest positive change in the PPLD occurring at rs1145787 (SL-PPLD = 0.71%, 2L-PPLD = 1.48%; see **Figure 5C**). While these numbers are very small, they are still considerably higher than the prior probability of LD, and viewed in terms of 2L–SL differences, rs1145787 is clearly salient.

In summary, SL linkage analysis in this single pedigree enabled us to narrow the primary genomic region of interest to 6 cM on chromosome 4, while fine-mapping based on LD within this region detected the true causal variant (locus 1) within this region along with one other variant in complete LD with the causal one. The modifier locus was not salient in the initial linkage scan, however, 2L analysis conditioning on genotype at locus 1 led to discovery of the true modifier variant. While both the PPL and the PPLD at this locus were relatively small, they were easily detected based on the amount of increase of the 2L signals relative to the original SL signals.

Kelvin can also be used to estimate the trait model using maximum likelihood estimators (m.l.e.’s) following the theory developed in Clerget-Darpoux et al. (1986), Elston (1989), Greenberg (1989), and Vieland and Hodge (1998). While our numerical integration routine is not optimized for maximization and therefore returns approximate rather than exact m.l.e.’s, it is interesting to note the models obtained from these analyses (**Table 2**). The disease allele frequency is estimated quite accurately by both PPL and PPLD analyses at locus 1; while at locus 2, the 2L-PPLD in particular returns an estimate reasonably close to the generating model. (Particularly at locus 2 where the PPL and PPLD themselves are quite low, the standard error of these estimates is likely to be substantial. Kelvin itself has no direct way to calculate these, but see Nouanesengsy et al., 2009 for further discussion.) More interesting, however, are the penetrance estimates. While there is no exact analog of the random reduced penetrance parameter of the SL model for a 2L generating model, using the approach described in Vieland et al. (1993), we obtain a SL penetrance vector “corresponding” to the generating model of (0.62, 0.62, 0) for the putative disease genotypes, respectively. This vector is approximated very closely by both the PPL and PPLD m.l.e.’s at locus 1. At the modifier locus, considering only individuals coded in the “dominant” LC based on locus 1, the estimated penetrance vectors indicate a virtually fully penetrant 2L dominant–dominant epistatic model. Thus overall, we were able not only to map both loci to the level of the individual variant, but also to determine the correct generating model with great accuracy, all in a single, highly informative pedigree.

**Table 2:** Approximate maximum likelihood trait parameter estimates.

Analysis	Locus	Disease allele frequency	Penetrances
SL-PPL	1	0.011	0.75, 0.56, 0.006
SL-PPLD	1	0.022	0.50, 0.49, 0.01
2L-PPL	2	0.125	0.99, 0.97, 0.011
2L-PPLD	2	0.25	0.99, 0.98, 0.011

*Penetrances are given for: (SL), D_1_D_1_, D_1_d_1_, and d_1_d_1_ genotypes, respectively, where “ D_1_” indicates the putative disease allele at locus 1; (2L) D_2_D_2_, D_2_d_2_, and d_2_d_2_ genotypes, respectively at locus 2, among those individuals who carry D_1_D_1_ or D_1_d_1_.*

### MCMC ACCURACY

**FIGURE 6:**
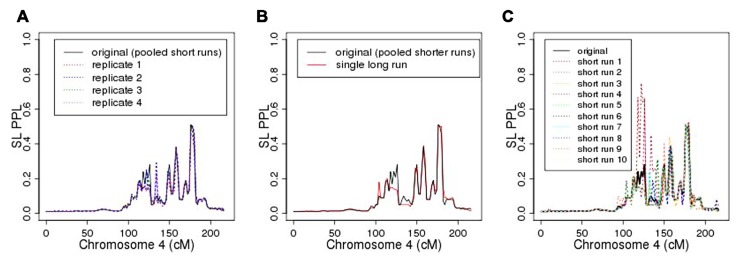
** (A)** Five replicates of chromosome 4 analysis; **(B)** original analysis compared to single long sampling run; **(C)** original (averaged) analysis compared to individual component short runs.

As seen in **Figure 6A**, repeating the entire MCMC sampling process five times produced very similar, albeit not identical, PPL profiles across chromosome 4. The marker log likelihood for chromosome 4 from the single long MCMC run still showed some upward convergence up to about 14,000 iterations, at which point it remained essentially flat. Comparing the final (post-convergence) 5,000 iterations with the original results (**Figure 6B**) again supported the accuracy of the original analysis in terms of the PPLs themselves. Again, however, the results are not identical. **Figure 6C** shows PPLs based on each of the component shorter sampling runs (as averaged over to obtain the original results) considered independently. There is considerable variation, particularly at positions further away from the true casual SNP. This strongly suggests, not surprisingly, that shorter runs of this length are not individually sufficient.

However, averaging across this set of shorter runs did enable us to achieve accurate results. Compared to a single, extremely long run, this is also a highly cost-effective approach insofar as it enables us to distribute the MCMC iterations to run concurrently on separate processors. On our hardware, the pooled-iteration process (using 10 servers with 2.5 GHz CPUs and 8 G memory) required 4 h, 40 min to complete chromosome 4, while the single long run (using one server) required 3 days, 5.5 h. Additional simulation studies are needed to further compare averaging across shorter sampling runs with use of single long sampling runs, especially across different pedigree structures with different patterns of missing data.

## DISCUSSION

We have illustrated an approach to gene discovery based on a single, highly informative family. This approach involves narrowing the genomic region(s) of interest using linkage analysis, followed by fine-mapping based on targeted LD (association) analysis in the same family. We have additionally illustrated how not just primary but even modifier genes can in principle be detected within a single pedigree.

Of course, we “cheated” by including the two causal SNPs in our association panel. In general, we might expect to have data from a standard SNP chip available on most family members for purposes of linkage and association mapping, together with sequence on a subset of individuals. In this case, the association analyses could be conducted on every observed sequence variant in the regions of interest, ensuring that the true mutation would be included (assuming that the relevant disease-causing element is a SNP). Of course to do this, the sequence variants would need to be measured in many family members, but at least in principle this could be done in part through imputation of sequence using sequencing in a subset of individuals combined with SNP data on the remaining individuals.

We chose our 2L generating model to be moderately mappable at the primary locus but with a modifier locus that was much harder to find. Of course in practice, realistic models may present more difficult challenges at all component loci, and this illustration is in no way to be construed as an estimate of any kind of power to find the genes. However, one salient feature of this approach is that it is not dependent on bioinformatic “filtering” approaches to prioritizing sequence variants as likely candidates. Instead, following the now classical reverse genetic paradigm, we rely entirely on positional mapping even at the variant-selection stage. Again, in practice this is likely to still leave a number of variants as candidates, since highly correlated variants under a peak may still be difficult to resolve statistically. Nevertheless, the number of such variants likely to be left on the list of candidates is greatly reduced by focusing on the linked and associated regions.

As noted above, the PPL framework is designed to measure strength of evidence, and not to test hypothesis or serve as a decision making algorithm. Thus at no point in the discussion did we consider “significance levels” or decide whether the evidence was “strong enough” to declare success. Rather, we relied on the accuracy of the framework overall as an evidence measurement technique, and simply followed up on the strongest evidence wherever that occurred. In this particular case, doing so led us to find both genes and both causal SNPs, without any “false positive” results. In practice, of course, difficult decisions would need to be made before, e.g., expending substantial resources following up functionally on the locus 2 SNP, given the very low PPLD. Nevertheless, had we set very stringent significance criteria from the outset and refused to follow-up on the strongest evidence regardless of the absolute numbers involved, we would have missed the modifier locus entirely. We note too that in consortium settings, Kelvin’s use of Bayesian sequential updating to accumulate evidence across data sets provides an alternative to traditional meta-analysis. Access to primary data, and not just summary measures such as *p*-values, is required for this. However, Kelvin outputs posterior marginal distributions, which can be used to sequentially update results across data sets without the need to actually pool the data themselves across sites.

The study design utilized here presented us with one salient computational challenge: how to compute the (parametric) likelihood for so large a pedigree. For this purpose we engineered a hybrid version of Kelvin using MCMC for the marker data and a non-stochastic method for integration over the trait parameters. This method proved to be quite accurate and computationally feasible, at least for data of this type. Of course the method can also be applied to sets of large pedigrees, and as noted, combined with ES- or LG-based analyses of smaller pedigrees or pedigrees with sparser marker maps for greater computational efficiency when analyzing data sets with variable family sizes.

Further studies in additional pedigree structures are needed to make specific recommendations regarding burn-in lengths and number of iterations needed to maximize the chances of accurate results for the MCMC portion of the calculation. In this regard, our new method is no better and no worse than McSample itself. However, we have some reason to think that the PPL and PPLD themselves may be relatively robust to some level of sampling variability in the underlying marker likelihood, possibly in part because integration over the trait model protects against modest amounts of imprecision at the marker level. This remains a subject for further investigation.

In this particular application, however, 2,000 samples derived from pooling the results of 10 independent sampling processes, each with 200 iterations following a 1,000-sample burn-in, appears to have been highly accurate. Still, this approach remains out of reach for genome-wide analysis on a typical desktop machine, requiring instead a distributed cluster environment to make real-time completion of results feasible. As high performance computing environments become more common for purposes of whole-genome sequence analysis and other “-omics” applications, we hope that this will become less of an impediment to analyses of the sort proposed here. Given the costs of data collection, we would argue that the additional computational demands are worth while if the methods are effective. The most definitive demonstration that they were effective in the current application is in the final results: successful mapping of two interacting disease loci down to the level of the individual causal variants.

## Conflict of Interest Statement

The authors declare that the research was conducted in the absence of any commercial or financial relationships that could be construed as a potential conflict of interest.
